# Ultralow-Thermal-Budget-Driven IWO-Based Thin-Film Transistors and Application Explorations

**DOI:** 10.3390/nano12183243

**Published:** 2022-09-19

**Authors:** Shanshan Jiang, Gang He, Wenhao Wang, Minmin Zhu, Zhengquan Chen, Qian Gao, Yanmei Liu

**Affiliations:** 1 School of Integration Circuits, Anhui University, Hefei 230601, China; 2 School of Materials Science and Engineering, Anhui University, Hefei 230601, China

**Keywords:** thin film transistor, low-temperature strategy, inverter, NAND logic, synaptic bionic

## Abstract

Exploiting multifunctional thin film transistors (TFTs) by low-temperature manufacturing strategy is a crucial step toward flexible electronics. Herein, a multifunctional indium–tungsten-oxide (IWO)-based TFT, gated by solid-state chitosan electrolyte membrane, is fabricated on paper substrate at room temperature. The chitosan exhibits a high specific electric-double-layer capacitance of 2.0 µF cm^−2^ due to the existence of mobile protons. The IWO-based TFT possesses excellent electrical properties, including a low threshold voltage of 0.2 V, larger current switching ratio of 1.3 × 10^6^, high field effect mobility of 15.0 cm^2^ V^−1^s^−1^, and small subthreshold swing of 117 mV/decade, respectively. Multifunctional operations including inverter, Schmitt triggers, and NAND gate are successfully demonstrated. As an example of information processing, the essential signal transmission functions of biological synapses also be emulated in the fabricated IWO-based TFTs. The experimental results indicate that such flexible IWO-based TFTs on low-cost and biodegradable paper provide the new-concept building blocks for flexible electronics.

## 1. Introduction

Nowadays, thin film transistors (TFTs) have been widely exploited and applied in optoelectronics, panel displays, electronic skins, and portable electronic products [1]. TFTs based on flexible substrates have attracted increasing attention owing to their unique advantages of flexibility, extensibility, and ultralight weight [2,3]. The continuous reduction in integration complexity and power consumption has accelerated the development of multifunctional flexible TFTs, which can simplify the device manufacturing process and circuit configuration [4]. Therefore, it is imperative to exploit flexible multifunctional TFTs with a low-temperature/cost manufacturing strategy and excellent electrical properties to meet the current market demands.

Many flexible materials, including thin glass, metal foils [5], plastic polymers [6], and paper [7], have been investigated as substrates in flexible electronic devices. Among them, paper has received much attention for flexible TFTs due to its recyclable, inexpensive, and biodegradable properties. Here, photo paper is selected as the substrate material for the low-cost fabrication of flexible TFTs. On the other hand, it is well known that the large capacitance of the gate dielectric is essential for low-voltage operations in TFTs. Solid-state electrolyte prepared at room temperature demonstrates a huge specific capacitance of >1.0 μF/cm^2^ when compared with conventional high-k gate dielectrics (such as HfO_2_, Al_2_O_3_, etc.) which usually need heat treatment [8]. Correspondingly, TFTs gating by solid-state electrolyte can induce an ultra-high-density carrier accumulation at a low operation voltage due to the electric double layer (EDL) electrostatic coupling at the electrolyte/semiconductor interface [9]. Among numerous electrolyte materials available for gate dielectric films, chitosan stands out because of its good film-forming property, high capacitance, high stability, low material cost, and easy preparation with an aqueous solvent. Chitosan, a natural electrolyte, is obtained by deacetylation of chitin. Such natural polymer is widely used in various industries for its bio-functionality and compatibility, blood compatibility, safety, and microbial degradability. Based on the above discussion, the chitosan membrane is selected as the gate dielectric material for the low-voltage and low-cost fabrication of flexible TFTs.

With the continuous exploration of semiconductor channel materials, metal oxide semiconductors are regarded as the potential channel layer candidates for integration into TFTs because of their high field effect mobility, low processing temperature, high optical transmittance, and good uniformity [10,11]. In recent years, amorphous indium gallium zinc oxide (a-IGZO) is one of the most interesting amorphous oxide semiconductor materials in scientific research and industrial application [12]. Unfortunately, TFT devices containing Ga/Zn active layer generally suffer from high humidity sensitivity, which is detrimental to the stability and performance of the devices [13,14]. Furthermore, heat treatment is a prerequisite for most IGZO-based TFTs to achieve superior device performances [15,16,17,18], which hinders their application in flexible electronics. Therefore, the development of non-annealed Ga/Zn oxide-free semiconductor materials becomes an essential technology for new-concept flexible TFT devices. Researchers have found that the amorphous indium–tungsten-oxide (a-IWO) semiconductor possesses higher bond dissociation and better acid resistance, which endows it with excellent stability and high mobility. Although several studies have been devoted to enhancing the electrical performance and stability of IWO-based TFTs [19], few efforts have been made to prepare the IWO channel layer at room temperature and integrate it with electrolyte. In this work, the annealing-free IWO thin film is selected as the channel layer combined with chitosan electrolyte for flexible TFTs with a low thermal budget.

Herein, ultralow-thermal-budget-driven IWO/chitosan flexible TFTs with high-performance and low-voltage operating are proposed for the first time on paper substrate. The field-effect mobility, current switching ratio, and subthreshold swing of TFTs are estimated to be 15.0 cm^2^ V^−1^ s^−1^, 1.3 × 10^6^, and 117 mV/decade, respectively. As a demonstration of potential multifunctional applications, a resistor-loaded inverter, Schmitt triggers, ‘NAND’ logic operation, and synaptic bionics are demonstrated experimentally. Such a flexible device is promising for low-cost, low-power consumption, and high-integration flexible electronic applications.

## 2. Materials and Methods

**Figure 1:**
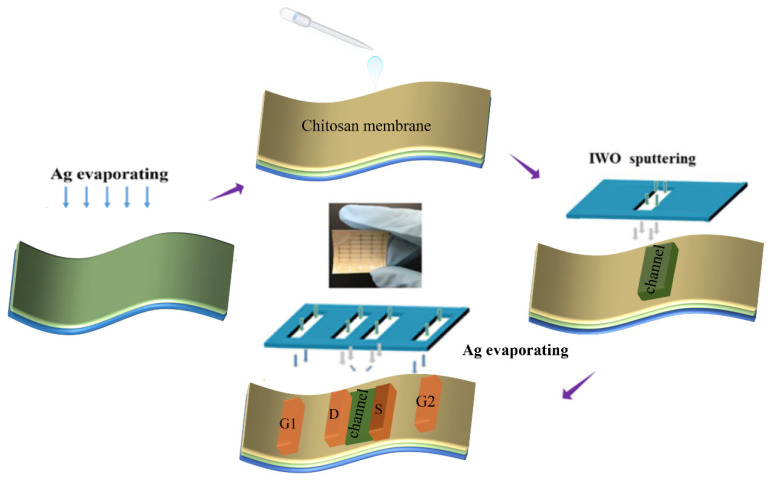
Fabrication process for flexible multi-gate IWO-based TFTs with chitosan gate dielectric on paper.

A schematic diagram of the preparation process of flexible multi-gate IWO-based TFTs is shown in Figure 1. A chitosan solution with the concentration of 2.0 wt% was prepared by dissolving chitosan powder (>99.5%, Aldrich, St. Louis, MO, USA) in deionized water. Prior to thin films deposition, the photo paper bought from Canon (Tokyo, Japan) was cleaned by a nitrogen gun to remove any impurities on the surface. Subsequently, Ag thin film was grown on photo paper by thermal evaporation as the bottom gate electrode. Then, the chitosan solution was spin-coated on Ag film and dried at room temperature to form a chitosan.

The solid-state electrolyte membrane had a thickness of about 15 µm. After that, the IWO channel layer with a thickness of about 20 nm was sputtered onto the chitosan layer using magnetron sputtering at room temperature. The IWO ceramic target with an In_2_O_3_: WO_3_ weight ratio of 99:1 was used. During the sputtering progress, the working power and the working pressure were 100 W and 0.5 pa, respectively. The channel width and length of the transistor are 1000 µm and 80 µm, respectively. Finally, the source/drain (S/D) and in-plane-gate (G1 and G2) electrodes were deposited by thermal evaporation. The electrical performances of IWO-based TFTs were measured by source measurement units (KEITHLEY 2636B and 2612 Source Meter, Cleveland, OH, USA).

## 3. Results and Discussion

**Figure 2:**
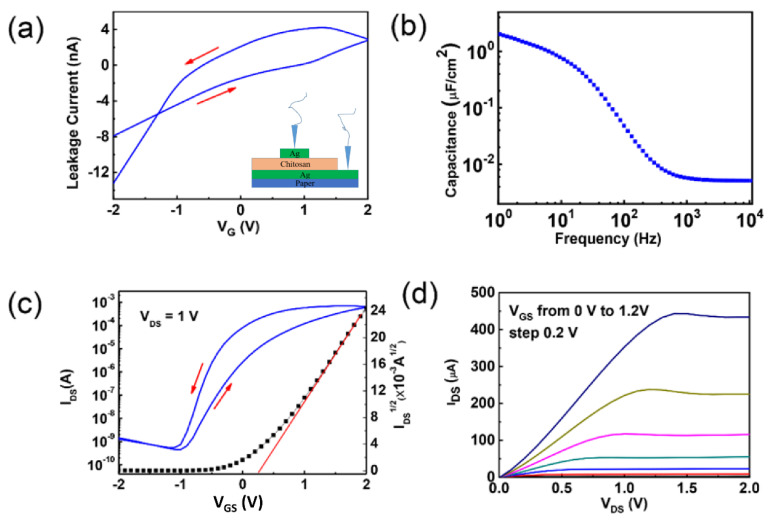
(**a**) Leakage current curve of the chitosan electrolyte membrane, the insert is the Ag/chitosan/Ag sandwiched testing structure; (**b**) Frequency-dependent specific capacitance curve of the chitosan gate dielectric film; (**c**) Transfer characteristic curve of the flexible IWO-based TFT; (**d**) Output characteristic curve of the flexible IWO-based TFT.

Figure 2a shows the leakage current curve of the chitosan gate dielectric film. The maximal leakage current is about 4 nA, which does not affect our device operated in a field-effect modulation model. Figure 2b exhibits the frequency-dependent specific capacitance curve of the chitosan electrolyte membrane. A huge specific capacitance of about 2 μF/cm^2^ can be observed at 1 Hz, which is due to the formation of EDL when applying an external electric field. Such high specific capacitance is favorable for the low voltage operation of the chitosan gated IWO-based TFTs. Figure 2c presents the transfer characteristic curves of the bottom-gate IWO-based TFTs gated by chitosan gate dielectric. As can be seen from the transfer characteristics, the maximum saturation current of the device is 9 × 10^−4^ A, and the minimum off-state current is 6.9 × 10^−10^ A. 

The current switching ratio I_on_/I_off_ of the device can be calculated to be 1.3 × 10^6^. The threshold voltage of the device can be extracted to be 0.2 V from the I_DS_^1/2^-V_GS_ curve, indicating that the prepared TFT is an enhanced device. The carrier mobility (μ) and subthreshold swing (SS) of IWO-based TFTs is calculated from the following equations, respectively:

(1) $$I_{\mathrm{DS}}=\frac{W}{L}\;\mu \;C_{i}\left[\left(V_{G}-V_{\mathrm{th}}\right)V_{\mathrm{DS}}-\frac{V_{\mathrm{DS}}{}^{2}}{2}\right]$$

(2) $$\mathrm{SS}=\left[\frac{d\left({\mathrm{logI}}_{D}\right)}{{\mathrm{dV}}_{G}}\right]$$

where I_DS_, C_i_, V_G_, and V_th_ are saturation drain current, areal capacitance, gate voltage, and threshold voltage, respectively. The mobility is calculated to be approximately 15.0 cm^2^ V^−1^ s^−1^and the sub-threshold swing is 117 mV/decade. Besides, it can be noted that there is a significant counterclockwise hysteresis in the transfer characteristic curve, which is due to the presence of protons in the chitosan film and the EDL coupling at the chitosan/IWO interface. Such behavior is essential for the realization of the neuromorphic functions [20]. From the electrical parameters extracted above, it can be concluded that the flexible IWO-based TFT possesses good electrical performance. As a comparison, Table 1 summarizes the electrical properties of different kinds of oxide-based TFTs gated by a chitosan gate dielectric. It can be clearly observed that the TFT based on the IWO semiconductor shows better performance than previously reported devices. Figure 2d shows the typical output characteristic curve of the IWO-based TFT with V_G_ varying from 0 V to 1.2 V. The output characteristic curve shows that the transistor exhibits good linearity when V_DS_ is small, and exhibits saturation when V_DS_ is large, which means that such a device has good ohmic contact and can be switched off properly. The maximum saturation current can reach 450 μA when V_DS_ = 2 V and V_Gs_ = 1.2 V.

**Figure 3:**
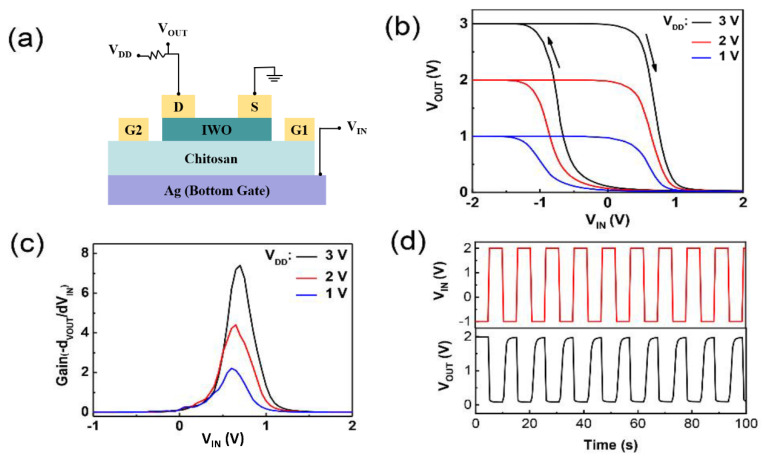
(**a**) Schematic illustration of resistor-loading inverter based on a flexible IWO-based TFT; (**b**) Voltage transfer characteristic curves; (**c**) Voltage gain characteristic curves; (**d**) Dynamic response behavior curves of the inverter.

To expand the potential logic application of flexible IWO-based TFTs, a low-voltage resistor-loading inverter is constructed by connecting an IWO-based TFT device in series with a resistor (2 MΩ) and connecting it to the supply voltage V_DD_. As shown in Figure 3a, the bottom gate electrode of the flexible TFT device is used as the input and the drain electrode as the output. Figure 3b shows the voltage transfer characteristics of the inverter at different bias voltages (V_DD_ = 1, 2, 3 V). Each characteristic curve exhibits good inverter behavior with a high level.

Output is equal to V_DD_ and a low level output equal to approximately 0 V [26], showing the good full-swing characteristics [27]. Figure 3c presents the voltage gain of the inverter, obtained by −∂V_OUT_/∂V_IN_; it is up to 7.4 at a supply voltage of 3 V, which is high enough to drive an integrated circuit with a large number of logic components. To test the repeatability of the flexible IWO-based TFT device, we input a pulse signal to the gate input terminal of the device with a low level of −1 V and a high level of 2 V. The dynamic response of the inverter is demonstrated in Figure 3d. When the input voltage is −1 V, the device can switch off and the drain output is high; while the input voltage is 2 V, the device can switch on and the drain output is low. The good repeatability of the response pulse indicates a high repeatability of the on-state current and current On/Off ratio. Besides, the low state of V_IN_ and V_OUT_ demonstrates that our device has potential application in low-cost paper electronics [28].

**Figure 4:**
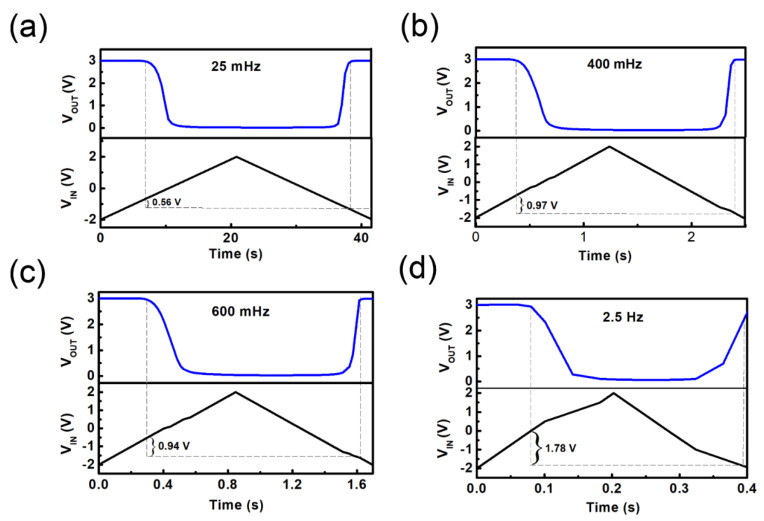
Transient response curve of the Schmitt triggers based on a flexible IWO-based TFT at driving frequencies of (**a**) 25 mHz, (**b**) 400 mHz, (**c**) 600 mHz, (**d**) 2.5 Hz.

A simple Schmitt triggers circuit can also be constructed based on the Schematic illustration in Figure 3a. Figure 4 shows the transient responses of the Schmitt triggers for different frequencies of triangular waveform input. It can be noted from Figure 4a–d that as the triangle wave frequency increases from 25 mHz to 2.5 Hz, the hysteresis window of Schmitt triggers increases from 0.5 V to 1.5 V. Besides, the on/off states and a variable hysteresis can be observed in this Schmitt triggers [20]. The changes in hysteresis window size of the Schmitt triggers can be attributed to the variation in the movement velocity of the protons in chitosan, and the frequency of the scanning voltage in the device under different frequencies of the input triangle wave. When the frequency of the input triangle wave increases, the proton movement speed of the device decreases, and the switching state of the Schmitt trigger will not be apparent.

**Figure 5:**
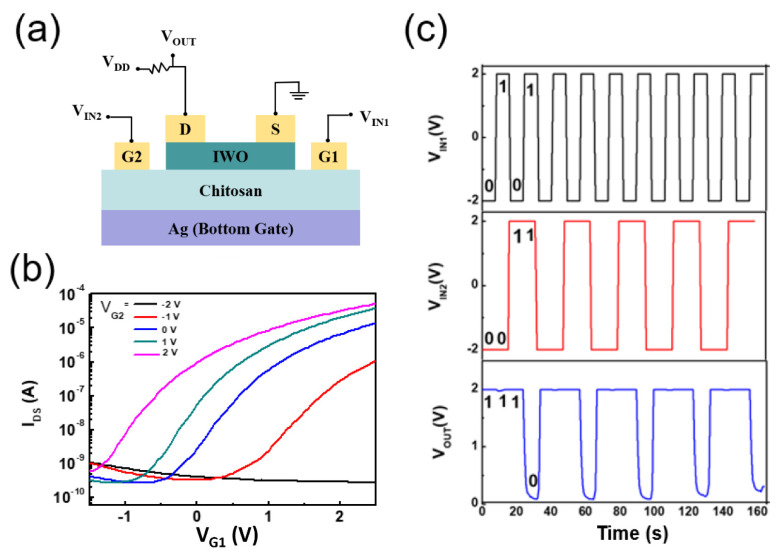
(**a**) Schematic of flexible multi-gate IWO-based TFT in series with a fixed resistor for performing the coordinated modulation and NAND gate; (**b**) Transfer characteristics curve of the TFTs measured with a sweep voltage applied on G1 and modulatory voltages applied on G2; (**c**) NAND logic demonstrated in flexible multi-gate IWO-based TFT.

In order to further realize multi-functional integration, the emerging multi-gate structure of flexible IWO-based TFTs is developed. Here, the laterally coupled dual-gate IWO-based TFTs will be explored to perform the coordinated modulation. To achieve this, the simple circuit configuration is shown in Figure 5a. The source of the flexible multi-gate IWO-based TFT is grounded, the drain is connected in series with a resistor, and then a supply voltage V_DD_ is connected. Two in-plane-gates G1 and G2 of the flexible multi-gate IWO-based TFT are used as input terminal and modulating terminal, respectively. The drain is regarded as the output terminal. The input voltage applied to gate G1 can be laterally coupled to the IWO channel layer via the EDL layer. After applying the modulating voltage to G2, it can be clearly seen from Figure 5b that the transfer characteristic curve of the flexible multi-gate IWO-based TFT can be effectively regulated by the voltage applied to the modulation gate terminal G2. For example, when V_G2_ is −2 V the TFT cannot be turned on even if the voltage applied to V_G1_ reaches 2 V. However, when V_G2_ increases, the TFT can be turned on easily and the source–drain current I_DS_ also increases with it. The modulating bias voltages on G2 are −2, −1, 0, 1, 2 V, respectively. The source–drain bias V_DS_ is always kept at 1 V. In such in-plane-gate IWO-based TFTs, we propose a model explained by the conductive layer of the bottom layer connected in series with two capacitors. The Ag conductive layer at the bottom of the transistor is regarded as the intermediate electrode, and the two capacitors formed at the interface between chitosan gate dielectric and Ag gate electrode can be connected in series through the Ag conductive layer. When V_G1_ > 0, electric double layer capacitor will be formed at the interface between the Ag electrode and the chitosan. While a negative voltage of −2 V is applied to V_G2_, most of the protons are bound in the vicinity of G2. It is almost impossible to form a current channel. However, as V_G2_ increases, the proton binding capacity decreases and a conductive charge is gradually present in the channel to turn the transistor on. When V_G2_ > 0, a capacitor that modulates the electrons in the IWO channel will form at the interface between the chitosan and Ag electrodes, thus allowing the gate to modulate the switching state of the transistor. 

Based on such flexible multi-gate IWO-based TFTs effectively modulating the source–drain current I_DS_ through additional modulating terminals, attempts have also been made to use them in logic circuits. As can be seen from the above, when the bias voltage of the regulated gate G2 is 2 V, the G1 bias effectively conducts or cuts off the drain current, and the device can also be turned on or off normally. In addition, the higher bias voltage of the regulation gate is, the easier the device is to turn on. With this type of access, the two inputs G1 and G2 can work together to determine whether the device can be turned on. Inputs G1 and G2 are defined as logic ‘1’ for high input level, and logic ‘0’ for low input level. The drain output is also defined as a logic ‘1’ when the output is high, and as a logic ‘0’when the output is low. The logic output-state ‘1’ can be obtained by importing the logic input-state ‘00’, ‘10’ or ‘01’, and the logic output-state ‘0’ is obtained by importing the logic input-state ‘11’. From the above, the flexible multi-gate IWO-TFT device can be connected to the circuit as in Figure 5a to achieve the function of a logical NAND gate. As shown in Figure 5c, NAND logic operations have been implemented. Due to the inherent limitations of EDL modulation of ion/proton migration, our devices operate slowly. Here we have implemented a NAND gate that operates at around 30 Hz. The NAND gate logic operation implemented in IWO-based TFT devices has a wide range of applications in artificial synapses and biosensors [29].

**Figure 6:**
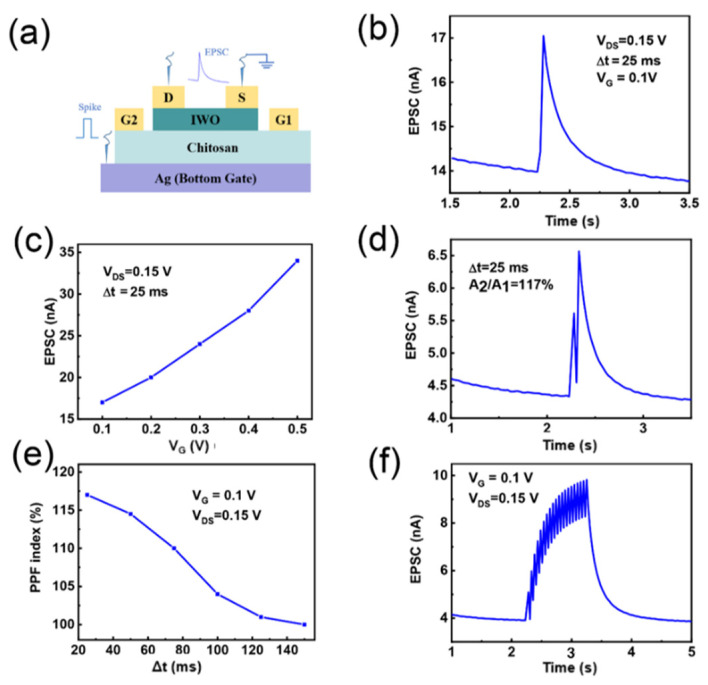
(**a**) Schematic of a flexible IWO-based TFT for realizing synaptic bionics; (**b**) The excitatory postsynaptic current (EPSC) triggered by a single pulse (0.1 V, 25 ms); (**c**) The maximum EPSC response current under different V_G_; (**d**) The paired-pulse responses triggered by paired-pulses (0.1 V, 25 ms); (**e**) The PPF index (A2/A1) as a function of the interval time Δt; (**f**) The EPSC response current triggered by 20 consecutive pulses (0.1 V, 25 ms) with a frequency of 20 Hz.

Finally, as a demonstration of information processing, the signal transmission of a biological synapse can be emulated by the flexible IWO-based TFTs. It is well known that pulses acting on presynaptic neurons can produce excitatory postsynaptic currents (EPSCs) in postsynaptic neurons. In this section, the bottom gate can be regarded as a presynaptic neuron, and the drain electrode D can be viewed as a postsynaptic neuron, as shown in Figure 6a. In order to emulate the single EPSC response, a pulse with an amplitude of 0.1 V and a width of 25 ms is applied at the bottom gate, and the source-drain voltage V_DS_ is kept at 0.15 V. The EPSC response that is similar to biological synapses can be observed in Figure 6b. Besides, increasing the voltage amplitude of the applied pulse will induce the enhancement of the maximum EPSC response, as shown in Figure 6c. The maximum EPSC response can be increased from 17 nA to 35 nA when V_G2_ increases from 0.1 V to 0.5 V. When two consecutive positive pulses (0.1 V, 25 ms) with an interval of 50 ms are applied to the bottom gate, it can be observed that the paired-pulse response current generated by the second pulse is larger than that generated by the first pulse, as shown in Figure 6d. Such behavior corresponds to the paired-pulse facilitation (PPF) phenomenon in biology, which can be described as the ratio of the second response amplitude A2 to the first response amplitude A1. PPF behavior is the essential short-term synaptic plasticity function. It participates in decoding temporal information in visual information processing, sound localization, and associative learning. A PPF index of 117% can be obtained by applying two consecutive pulses with an interval of 25 ms to the flexible IWO-based TFT. This behavior is mainly attributed to the accumulation of charge carriers caused by the slow retention of protons. When the time interval between two consecutive pulses is increased, the PPF index will obviously decrease, as shown in Figure 6e. Such phenomena can be easily explained by the competitive effect between proton accumulation and diffusion effect.

When the time interval between two pulse increases the time for protons to diffuse back to the original equilibrium state becomes enough, so the weak accumulation effect and the decreasing facilitation rate are obtained. Besides, continuous accumulation of EPSC responses can also be realized. By applying a pulse sequence to the bottom gate of the flexible IWO-based TFT, it can be observed from Figure 6f that the EPSC response current gradually increases. The pulse sequence with a frequency of 20 Hz contains 20 pulses (0.1 V, 25 ms). Here, the EPSC gain can be defined as the ratio of the amplitude of the twentieth EPSC peak (A20) to the first EPSC peak (A1) at the frequency of 20 Hz. Namely, the prepared multifunction IWO-based TFT can increase the EPSC gain to 200%.

## 4. Conclusions

In this work, the development strategy for obtaining multifunctional and high-performance flexible TFTs with low-thermal-budget and low-cost has been successfully realized. Flexible multifunctional IWO-based TFTs gated by chitosan gate dielectric were fabricated on paper substrates at room temperature. Such TFTs operated at low voltage exhibited high electrical performances, including a larger current switching ratio of 1.3 × 10^6^, high field effect mobility of 15 cm^2^ V^−1^ s^−1^, and small sub-threshold swing of 117 mV/decade, respectively. A resistor-loaded inverter based on the flexible IWO-based TFT showed good full-swing characteristics, and its voltage gain is up to 7.4. A simple Schmitt trigger and NAND logic operation were also successfully demonstrated. In addition, essential synaptic functions such as EPSC and PPF behaviors can be emulated in the flexible IWO-based TFT. Undoubtedly, such a device would provide a reasonable road for the next generation of flexible electronics with low-power consumption and high-integration.

## Figures and Tables

**Table 1:** Recent advances in oxide-based TFTs gated by a chitosan gate dielectric.

Channel Layer	SS [V/Decade]	Mobility [cm^2^V^−1^s^−1^]	I_on_/I_off_	V_th_ [V]	References
ITO	0.095	3.6	~10^5^	0.02	[21]
ITO	0.093	5.7	~10^6^	−0.05	[22]
IGZO	-	3.3	5.1 × 10^5^	0.57	[23]
ZnO	0.135	7.8	10^5^	1.00	[24]
ITO	0.091	5.6	10^7^	0.10	[25]
IWO	0.117	15.0	1.3 × 10^6^	0.20	This work

## Data Availability

Not Applicable.
